# An epigenetic map of age-associated autosomal loci in northern European families at high risk for the metabolic syndrome

**DOI:** 10.1186/s13148-015-0048-6

**Published:** 2015-02-20

**Authors:** Omar Ali, Diana Cerjak, Jack W Kent, Roland James, John Blangero, Melanie A Carless, Yi Zhang

**Affiliations:** Department of Pediatrics, Medical College of Wisconsin, Milwaukee, Wisconsin USA; TOPS Obesity and Metabolic Research Center, Department of Medicine, Medical College of Wisconsin, Milwaukee, Wisconsin USA; Human and Molecular Genetics Center, Medical College of Wisconsin, 8701 Watertown Plank Road, Milwaukee, WI 53226, Wisconsin USA; Department of Genetics, Texas Biomedical Research Institute, San Antonio, Texas USA

**Keywords:** Age, Epigenetics, MetS, T2D, Family study

## Abstract

**Background:**

The prevalence of chronic diseases such as cancer, type 2 diabetes, metabolic syndrome (MetS), and cardiovascular disease increases with age in all populations. Epigenetic features are hypothesized to play important roles in the pathophysiology of age-associated diseases, but a map of these markers is lacking. We searched for genome-wide age-associated methylation signatures in peripheral blood of individuals at high risks for MetS by profiling 485,000 CpG sites in 192 individuals of Northern European ancestry using the Illumina HM450 array. Subjects (ages 6–85 years) were part of seven extended families, and 73% of adults and 32% of children were overweight or obese.

**Results:**

We found 22,122 genome-wide significant age-associated CpG sites (*P*_α=0.05_ = 3.65 × 10^−7^ after correction for multiple testing) of which 14,155 are positively associated with age while 7,967 are negatively associated. By applying a positional density-based clustering algorithm, we generated a map of epigenetic ‘hot-spots’ of age-associated genomic segments, which include 290 age-associated differentially methylated CpG clusters (aDMCs), of which 207 are positively associated with age. Gene/pathway enrichment analyses were performed on these clusters using FatiGO. Genes localized to both the positively (*n* = 241) and negatively (*n* = 16) age-associated clusters are significantly enriched in specific KEGG pathways and GO terms. The most significantly enriched pathways are the hedgehog signaling pathway (adjusted *P* = 3.96 × 10^−3^) and maturity-onset diabetes of the young (MODY) (adjusted *P* = 6.26 × 10^−3^) in the positive aDMCs and type I diabetes mellitus (adjusted *P* = 3.69 × 10^−7^) in the negative aDMCs. We also identified several epigenetic loci whose age-associated change rates differ between subjects diagnosed with MetS and those without.

**Conclusion:**

We conclude that in a family cohort at high risk for MetS, age-associated epigenetic features enrich in biological pathways important for determining the fate of fat cells and for insulin production. We also observe that several genes known to be related to MetS show differential epigenetic response to age in individuals with and without MetS.

**Electronic supplementary material:**

The online version of this article (doi:10.1186/s13148-015-0048-6) contains supplementary material, which is available to authorized users.

## Background

Chronic diseases such as cancer, type 2 diabetes (T2D), metabolic syndrome (MetS), cardiovascular disease, and dementia constitute the most common health problems seen in developed societies (and increasingly, in developing societies), and their prevalence increases with age in all populations [1-4]. It is well established that environmental exposures, especially in early life, can alter the risk of various chronic diseases later in life [5,6], and while the mechanisms involved in this ‘programming’ of future risk are not yet understood in detail, epigenetic changes are believed to play an important role in this process [7,8].

Epigenetic mechanisms mediate the interaction between gene and environment throughout the lifespan; while the underlying genetic sequence does not change, environmental influences can alter epigenetic marks and thus alter gene expression and induce long-term changes in phenotype and disease susceptibility [9]. The gradual accumulation of epigenetic changes in critical genes may contribute to the observed age-related increase in the prevalence of various chronic disorders [10-13]. Epigenetic changes are known to be heritable across more than one generation of offspring in plants and mammals [14-19], and there is evidence that transgenerational epigenetic inheritance also occurs in humans [20-23]. Such transgenerational inheritance of epigenetic states may contribute to the observed inherited risk of various chronic disorders, including metabolic disorders [24].

DNA methylation is one of the most extensively studied epigenetic mechanisms and plays an important role in the process of development and differentiation [25]. There is evidence from both human and animal sources that prenatal nutritional deprivation can permanently alter DNA methylation at multiple loci, and these changes play a role in the observed alteration of future risk of chronic diseases like obesity, insulin resistance, and diabetes [26-32]. It is also known that DNA methylation patterns continue to change after birth, at least partly in response to environmental influences [33-35]. For example, studies show that identical twins have broadly similar epigenetic profiles *in utero* but these profiles gradually diverge as they get older [36-38]. Several studies have looked at the effect of aging on genome-wide DNA methylation in adults, and these studies show that age-dependent methylation changes are found in a variety of tissues and correlate well enough with age that the methylation status of selected loci can be used to predict the age of a subject [35,39-41].

These age-related methylation changes may play a role in the observed age-related risk of various chronic diseases. For example, studies show that the hyper-methylation of certain CpG loci is associated with increased cancer risk via reduced expression of cancer-suppressor genes [41,42]. It has been proposed that age-related changes in DNA methylation play a similar role in increasing the risk of obesity, T2D, and MetS, but the specific genes involved and the specific changes in their functioning are yet to be determined [43-45].

Studies of genome-wide DNA methylation can be conducted using various populations, and each design has its advantages and disadvantages. For example, monozygotic twins are genetically identical, so epigenetic differences found in twin pairs are likely to be either stochastic or environmentally induced, rather than genetically inherited [46]. In studies using both mono- and dizygotic twins, it is possible to estimate the relative effect of genetic versus environmental influences to some extent. But since twin pairs are of the same age, the differential effect of age cannot be compared within the pairs and the comparison of twins of different ages does not offer any special advantage over comparing two unrelated individuals of different ages. Studies using unrelated subjects have the advantage that large numbers of subjects are relatively easy to recruit, but the epigenetic landscape of unrelated subjects can be influenced by population structure and systematic differences in environmental exposures that may not be easy to identify and that may thus confound the results. In a family-based cohort, we can reduce the confounding effect of genetic variation and population structure and, when large extended families live close to each other and follow similar traditions, they share many environmental factors as well, thus reducing the confounding effect of group differences such as in diet and geographical location. Because such family-based cohorts include related individuals of different ages in the same cross-sectional study, it is possible to examine changes associated with age against a relatively stable genetic and environmental background. Another advantage of using large extended pedigrees is that it may be possible to identify epigenetic patterns that are associated with disease risk specifically within that family and not in the general population. A family-based cohort can thus be an especially powerful tool for identifying age-related methylation changes, including changes that are universally associated with aging and those that are specific to families with shared genetic and environmental risk factors for particular chronic diseases.

While no epigenome-wide study of extended, multi-generational families has yet been published, a recent study on a combination of twin and their nuclear family members examined the role of genetic features on DNA methylation [23]. These authors suggest that the majority of transgenerational similarity in DNA methylation can be explained by shared genetic effects and that epigenetic inheritance (incomplete erasure of epigenetic modifications across generations) has a relatively limited role in the observed inherited risk of various chronic disorders. This observation still needs to be confirmed in other studies and in populations at high risk for particular chronic disorders.

We assembled a cohort comprising several large extended families of Northern European descent that is enriched for obesity, central adiposity, and obesity-associated MetS traits. To identify genomic regions whose methylation status changes with aging, we conducted a genome-wide survey of peripheral blood DNA methylation and interrogated more than 485,000 CpG sites in 192 subjects from seven extended families living in two US Midwestern states, Wisconsin and Illinois.

## Results

### The TOPS family study of epigenetics

The TOPS Family Study of Epigenetics (TFSE) was designed to study the role of epigenetic mechanisms in linking genes and the environment using related subjects of large extended pedigrees. The average age of the cohort is 36.2 (±18.8) years, 28% of the subjects were 18 years and younger at ascertainment and 55% are females. As the subjects were selected from families that are part of a previous genetic study on the metabolic risk complications of obesity [47], the cohort is enriched for obesity and MetS traits (Table 1) with 73% of the adults being overweight or obese (based on body mass index (BMI)), 52% with waist circumference above MetS thresholds (>102 cm in men; >88 cm in women), 31.9% with evidence of insulin resistance (based on homeostatic model of assessment (HOMA) > 3.5) [48,49], 20.3% with hypertriglyceridemia (>150 mg/dl), and 65.7% with high-density lipoprotein (HDL) below MetS thresholds (<40 mg/dL in males and <50 mg/dl in females). Overall, 23.7% of the adults of our cohort met the ATPIII definition of having MetS [50]. In the pediatric subjects, the prevalence of overweight and obesity (BMI >85th percentile) was 32%. All analyses accounted for the relatedness of family members by conditioning the fixed effects of methylation status on the expected genetic similarity of relatives (Table 2) [51].

**Table 1:** **TOPS Family Study of Epigenetics (TFSE) cohort characteristics**

	**Children and adolescents**		**Adults**	
	**(mean ± SD)**		**(mean ± SD)**	
Overweight and obese	32%		73%	
Waist over MetS threshold	15%		52%	
HOMA over MetS threshold	17%		32%	
Hypertriglyceride	9%		20%	
HDL-C over MetS threshold	51%		66%	
MetS prevalence	NA		24%	
Phenotype	Girls (*n* = 21)	Boys (*n* = 32)	Female (*n* = 85)	Male (*n* = 54)
Weight, kg	56.35 ± 22.97	58.53 ± 25.15	82.98 ± 19.63	94.60 ± 23.36
Height, cm	157.81 ± 13.09	159.69 ± 20.93	164.62 ± 6.87	177.31 ± 6.38
BMI, kg/m_2_	22.10 ± 7.50	21.80 ± 5.70	30.75 ± 7.66	30.04 ± 6.76
BMI%	55.61 ± 34.20	66.91 ± 26.16	NA	NA
Waist circumference (WC), cm	68.15 ± 14.05	75.16 ± 18.15	93.87 ± 17.89	101.58 ± 16.92
Subcutaneous fat (SubQF), g	361.49 ± 263.70	123.31 ± 95.00	319.34 ± 191.59	255.94 ± 131.42
Visceral fat (VF), g	78.57 ± 24.02	43.66 ± 25.27	170.84 ± 149.50	265.24 ± 94.95
VF/SubQF	0.32 ± 0.20	0.45 ± 0.20	0.56 ± 0.32	1.13 ± 0.40
Total abdominal fat (TAF), g	440.05 ± 287.49	166.97 ± 114.39	490.18 ± 300.40	521.18 ± 204.41
Fasting glucose (FG), mmol/l	82.28 ± 8.44	86.47 ± 8.54	84.26 ± 13.07	86.58 ± 15.37
Fasting insulin (FI), pmol/l	14.81 ± 5.50	13.73 ± 5.69	16.12 ± 15.67	21.07 ± 22.91
Insulin/glucose (IGR)	0.18 ± 0.06	0.16 ± 0.07	0.44 ± 1.66	0.52 ± 1.42
Homeostasis model assessment insulin resistance (HOMA-IR)	3.03 ± 1.22	2.94 ± 1.32	3.53 ± 3.79	4.69 ± 5.15
Triglycerides (TG), mmol/l	78.52 ± 28.11	78.00 ± 45.44	100.07 ± 52.44	117.69 ± 62.38
Total Cholesterol (TC), mmol/l	152.76 ± 22.71	142.66 ± 25.78	193.06 ± 41.21	194.61 ± 38.77
LDL-cholesterol (LDL-C), mmol/l	89.29 ± 24.62	85.75 ± 23.15	125.91 ± 38.39	133.82 ± 36.28
HDL-cholesterol (HDL-C), mmol/l	46.95 ± 9.93	41.31 ± 9.50	46.29 ± 13.46	37.68 ± 9.93
Systolic blood pressure (sBP), mmHg	105.50 ± 5.00	107.55 ± 10.42	127.34 ± 18.56	129.61 ± 13.97
Diastolic blood pressure (dBP), mmHg	66.94 ± 8.30	69.45 ± 9.59	76.79 ± 10.89	81.94 ± 11.39
Adiponectin, ng/ml	14.42 ± 5.96	10.45 ± 5.01	9.67 ± 5.52	7.95 ± 6.29
Leptin, ng/ml	13.46 ± 11.26	6.66 ± 7.17	23.72 ± 14.15	9.85 ± 7.22

**Table 2:** **Pair-wise relationships within TFSE pedigrees**

**Number of relative pairs**	**Familial relationship**	**Proportion of alleles shared IBD**
196	Parent-offspring	1/2
212	Siblings	1/2
91	Grandparent-grandchild	1/4
439	Avuncular	1/4
1	Half siblings	1/4
13	Great grandparent-grandchild	1/8
275	Grand avuncular	1/8
2	Half avuncular	1/8
454	First cousins	1/8
598	First cousins, 1 rem	1/16
1	Half first cousins	1/16
236	Second cousins	1/32

*IBD* identical by descent.

### The genome-wide autosomal map of age-associated DNA methylation in the TFSE cohort

We have implemented a data cleaning procedure aiming to retain only the informative CpG probes for downstream analyses (see Additional file 1). Of a total of 485,512 CpG sites that were assayed on the Illumina HM450 panel, a total of 137,168 autosomal CpG sites passed our data cleaning procedure and were entered into our statistical analysis pipeline for age association tests.

**Figure 1:**
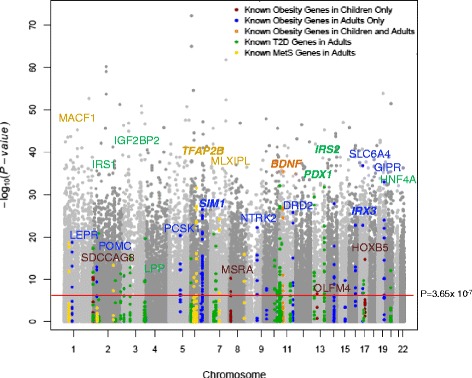
**Strength of associations of genome-wide autosomal CpG methylation status with age in our TFSE cohort.** Manhattan plot shows the significance level of age association of each CpG locus by chromosomal position. Each grey dot represents an individual CpG site. The red line depicts the genome-wide significance threshold after correction for Bonferroni multiple testing, *P*_α=0.05_ = 3.65 × 10^−7^. Colored dots represent epigenetic regions belonging to genes previously associated with obesity, T2D, and MetS. *Blue dots* obesity-related genes in adults, *red dots* obesity related genes in children, *orange dots* obesity related genes in children and adults, *green dots* T2D genes, *yellow dots* MetS genes. Selected genes are labeled. Genes found to be within an aDMC are shown in bold and italicized.

**Figure 2:**
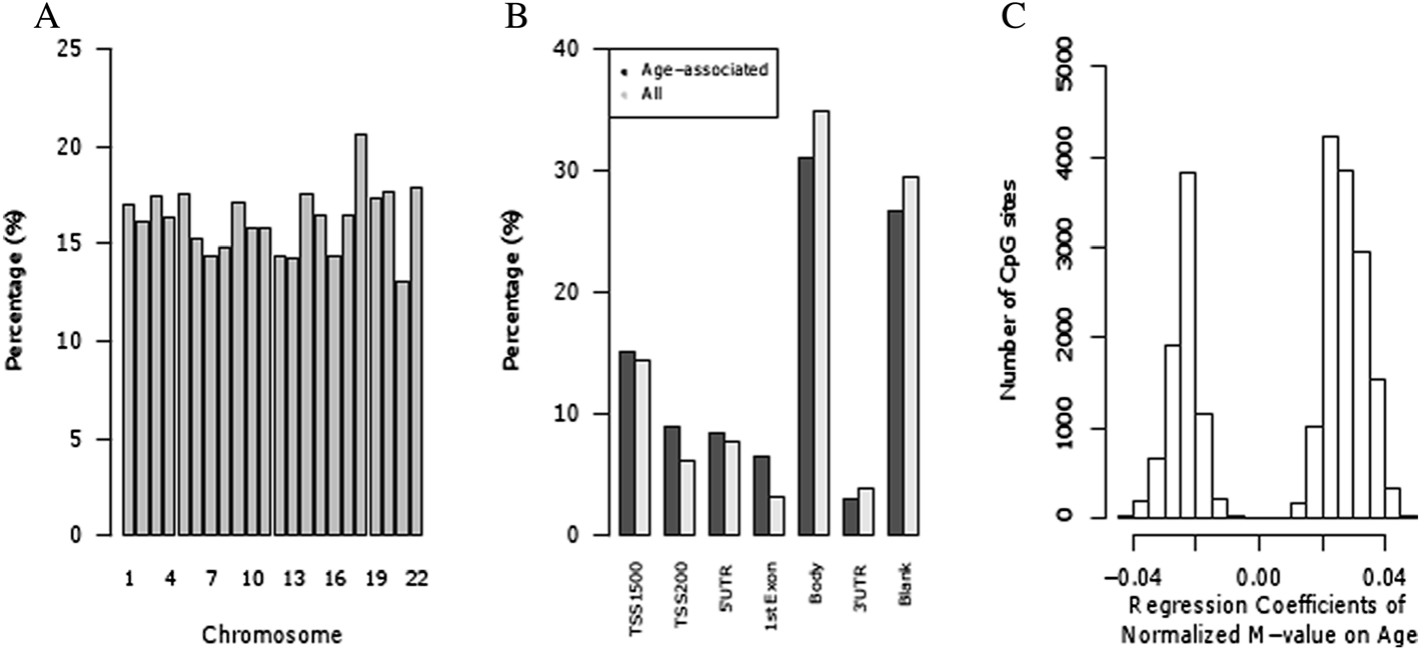
**Characteristics of age-associated CpG sites. (A)** Methylation sites are shown based on their chromosomal location (*x*-axis). The percentage of sites was determined by the number of age-associated sites on each chromosome over the number of sites in the analysis on each chromosome (*n* = 137,168). **(B)** Histogram shows the distribution of genome-wide age-associated sites in relation to gene architecture. ‘Age-related’ sites are the 22,122 sites that were found to be genome-wide age-associated and ‘All’ are the locations of all the sites run in the analysis (*n* = 137,168). **(C)** Histogram of regression coefficients for 22,122 loci with genome-wide significant association with age.

**Figure 3:**
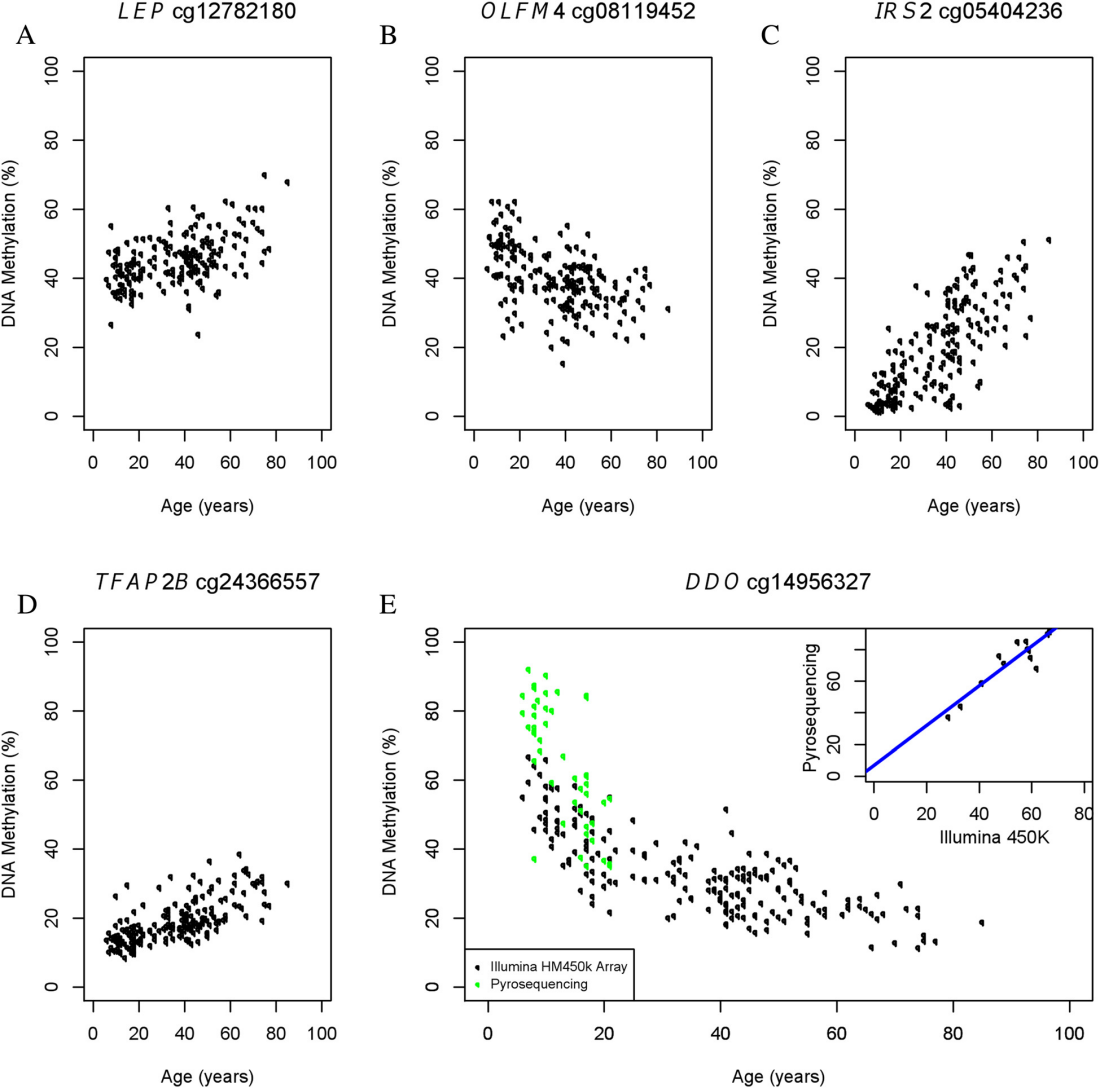
**DNA methylation changes with age in candidate genes related to obesity, MetS, and T2D. (A)** Methylation of CpG site cg12782180 located in the TSS1500 of *LEP* with respect to age. **(B)** Methylation of CpG site cg08119452 located in the TSS1500 of *OLFM4* with respect to age. **(C)** Methylation of CpG site cg05404236 located in the first exon of *IRS2* with respect to age. **(D)** Methylation of CpG site cg24366557 located in the body of *TFAP2B* with respect to age. **(E)** Pyrosequencing validation of differential methylation with age identified by arrays. Scatter plot shows the overlay of age-associated methylation changes at CpG site cg14956327 (*DDO*) probed by the Illumina HM450 array (black dots) and pyrosequencing (green dots). Only children and adolescents samples were used in validation tests. The insert shows direct comparisons between the Illumina array and pyrosequencing validation.

Using methylation status represented by *M* values (see the ‘Methods’ section), we tested each epigenetic marker for association with age in linear mixed models that included the random effect of kinship using SOLAR [52] (see the ‘Methods’ section). Our models also accounted for the fixed effects of sex and blood cell subtype proportions. Figure 1 shows a Manhattan plot of the CpG sites whose methylation status was associated with age. Of these, 22,122 age-associated CpG sites in our cohort surpassed the genome-wide significance threshold (*P*_α=0.05_ = 3.65 × 10^−7^ after correction for multiple testing). The characteristics of these age-associated CpG sites are shown in Figures 2 and 3. The percentage of genome-wide age-associated sites per probes on each chromosome is shown in Figure 2A. We observed that 39% of these age-associated sites are located within potential regulatory regions of genes (from 5′ UTR to the first exon, Figure 2B). The effect of age on DNA methylation at each individual CpG site is shown as regression coefficients of normalized *M* value per year of age in Figure 2C. We found that 14,155 of these genome-wide significant CpG sites are positively associated with age while 7,967 sites are negatively associated. This gives a ratio of 1.8 for epigenetic loci that exhibit increasing methylation over time versus those that show decreasing methylation. Examples of genome-wide significant age-associated sites include CpG loci located in the promoter region of the obesity gene *LEP* [53], the childhood obesity gene *OLFM4* [53], the T2D gene *IRS2* [54], and the newly identified MetS gene *TFAP2B* [55] (Figure 3A–D).

To validate the methylation results obtained using the HM450 array, we quantitatively assessed the methylation status of CpG locus cg14956327 in 48 samples ranging from 6–21 years of age using pyrosequencing (Figure 3E). The graph within Figure 3E also shows a direct comparison of 13 samples between the Illumina array and pyrosequencing validation.

We also assessed the coverage of our data by comparing our study to several recent studies of age-associated epigenetic markers (Additional files 2 and 3). We found that the current study has an exceptionally broad age range (with subjects ranging from 6 to 85 years in age) and was able to identify from 38% to 100% of previously identified age-associated CpG sites depending on the study in comparison. For example, we have identified significant age-associations at CpG sites in the genes *ELOVL2*, *FHL2*, *KLF14*, and *PENK* that had been repeatedly reported as strongly age-associated methylation markers (Additional file 3) [39,56,57]. Our study also identified 21,078 additional genome-wide significant CpG sites that have not been reported in these previous studies.

### Bioinformatic analysis of genome-wide significant age-associated DNA methylation sites in the TFSE cohort

We hypothesized that in our subjects, whose family history (genetic and environmental) makes them susceptible to developing obesity and MetS, age-associated DNA methylation is enriched in genes and pathways involved in metabolic homeostasis. Changes caused by aging in these epigenetic states might lead to the malfunctions that underlie the increased prevalence of clinical symptoms of obesity and MetS in the aging population [1,3]. As the subjects we interrogated for genome-wide DNA methylation profiles were from families at high risk for developing MetS, we looked at genes with prior evidence for involvement in obesity and T2D and checked if there were CpG sites above genome-wide significance level associated with any of them.

#### Known genes for obesity

We found that 20 genes out of the 36 listed in a gene list based on genetic studies of human obesity [53] have one or more methylation sites that is significantly associated with age in our families (Figure 1, Additional file 4). These genes include a number of well-studied obesity genes such as *LEP*, *POMC*, *PPARG*, and *CNR1*, as well as previous obesity GWAS candidates with unclear roles in obesity etiology, such as *SIM1*, *IRX3*, and *SLC6A11*. Furthermore, four genes identified in GWAS for childhood obesity, such as *SDCCAG8*, *TNKS*/*MSRA*, *OLFM4*, and *HOXB5*, were found to be epigenetically age-associated as well.

#### Genes known to be associated with type 2 diabetes

Of the 20 T2D susceptibility loci recently identified in GWAS [58], ten were found to be epigenetically modified by age in our analysis (Figure 1, Additional file 5). These include *PPARG*, *HNF1B*(*TCF2*), *TCF7L2*, *IGF2BP2*, *HHEX*/*IDE* , *KCNQ1*, *MTNR1B*, *ADAMTS9*, *THADA*, and *JAZF1*. In one of the most recent studies of T2D genes using genome-wide trans-ancestry meta-analysis, seven novel loci were identified [59]. Of these seven loci, we found evidence for age-associated differential methylation at *SSR1*/*RREB1* and *LPP*. We also looked for any age-associated epigenetic evidence for genes established through approaches other than GWAS and found that the following known T2D genes are under epigenetic regulation by age: *IRS1* [60], *IRS2* [54], *AKT* [61], *ABCC8* [62], *HNF4A* [63], *IPF*-1(*PDX1*) [64], *NeuroD1* [65], and *GCK* [66].

#### Pleiotropic genes known to be associated with MetS

Of the 25 genes that have been shown to play pleiotropic roles in MetS and inflammation [55], we found eight genes with one or more methylation sites significantly associated with age in our families (Figure 1; Additional file 6). These genes include *GRB14*, *KIAA0754*, *MACF1*, *MLXIPL*, *SKIV2L*, *STK19*, *TFAP2B*, and *TRIB1.*

### The map of genomic locations of dense age-associated differentially methylated clusters in the TFSE cohort

**Figure 4:**
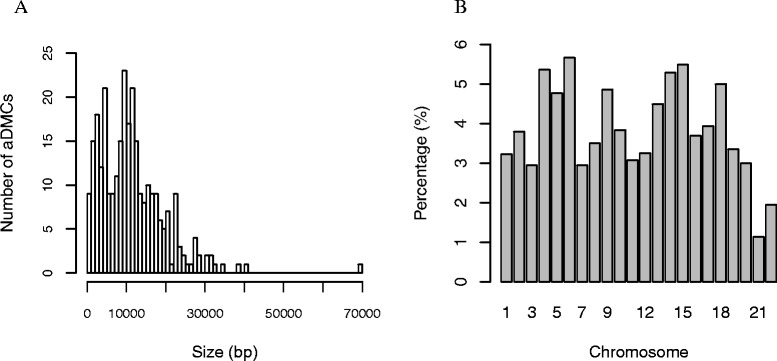
**Characteristics of aDMCs defined in our cohort. (A)** Histogram shows the size distribution (bp) of the 290 aDMCs. **(B)** aDMCs are shown based on their chromosomal location (*x*-axis). The percentage of aDMCs was determined by the number of aDMCs on each chromosome over total number of aDMCs on each chromosome (*n* = 7,590).

We then applied a modified ‘bump-hunting’ algorithm to identify clusters of age-related methylated CpG sites. Using this algorithm, we generated a map of epigenetic ‘hot-spots’ of age-associated genomic segments. In our search algorithm, modified from a previously published method [67,68], we defined a group of sites as a dense age-associated CpG cluster when at least 50% of no less than ten sites are associated with age at genome-wide significance, and the distance between any pair of age-associated sites is no greater than 10 kb. Additional file 7 shows the autosomal map of age-associated differentially methylated CpG clusters (aDMCs) throughout the autosomal genome. We identified 290 aDMCs, of which 207 are positively associated with age, 9 are negatively associated, and 74 have sites associated in either direction. The detailed characteristics of these identified aDMCs are depicted in Figure 4. The distribution of the sizes of these aDMCs showed one peak in the aDMCs with sizes around 5 kb and another around 10 kb (Figure 4A). To study the chromosomal distribution of these aDMCs, we divided the total number of age-associated clusters on each chromosome by the total number of clusters that would be generated if the same algorithm was applied to all loci that survived our data cleaning procedure. We found that 3.8% of all clusters generated by this algorithm are age-associated and they are mostly evenly distributed across the genome, with a few chromosomes being modestly over-represented (Figure 4B). The sizes of identified aDMCs range from 457 to 69,237 bp, and we found that 80 of these aDMCs span over more than one gene (up to a maximum of four genes). When we filter genes known for obesity, T2D, and MetS based on our clustering criteria, three obesity genes, two T2D genes, and one MetS gene each contains at least one aDMC. For instance, we found an aDMC that spans a 2.1-kb region of the gene insulin receptor substrate 2 (*IRS2*) on chromosome 13. This cluster begins within the promoter region (1,500 bp before transcription starting site (TSS)), 5′ untranslated region (UTR) and ends in the first exon (Figure 1; Additional file 5).

### Gene network and pathway analyses of aDMCs in our family cohort

**Figure 5:**
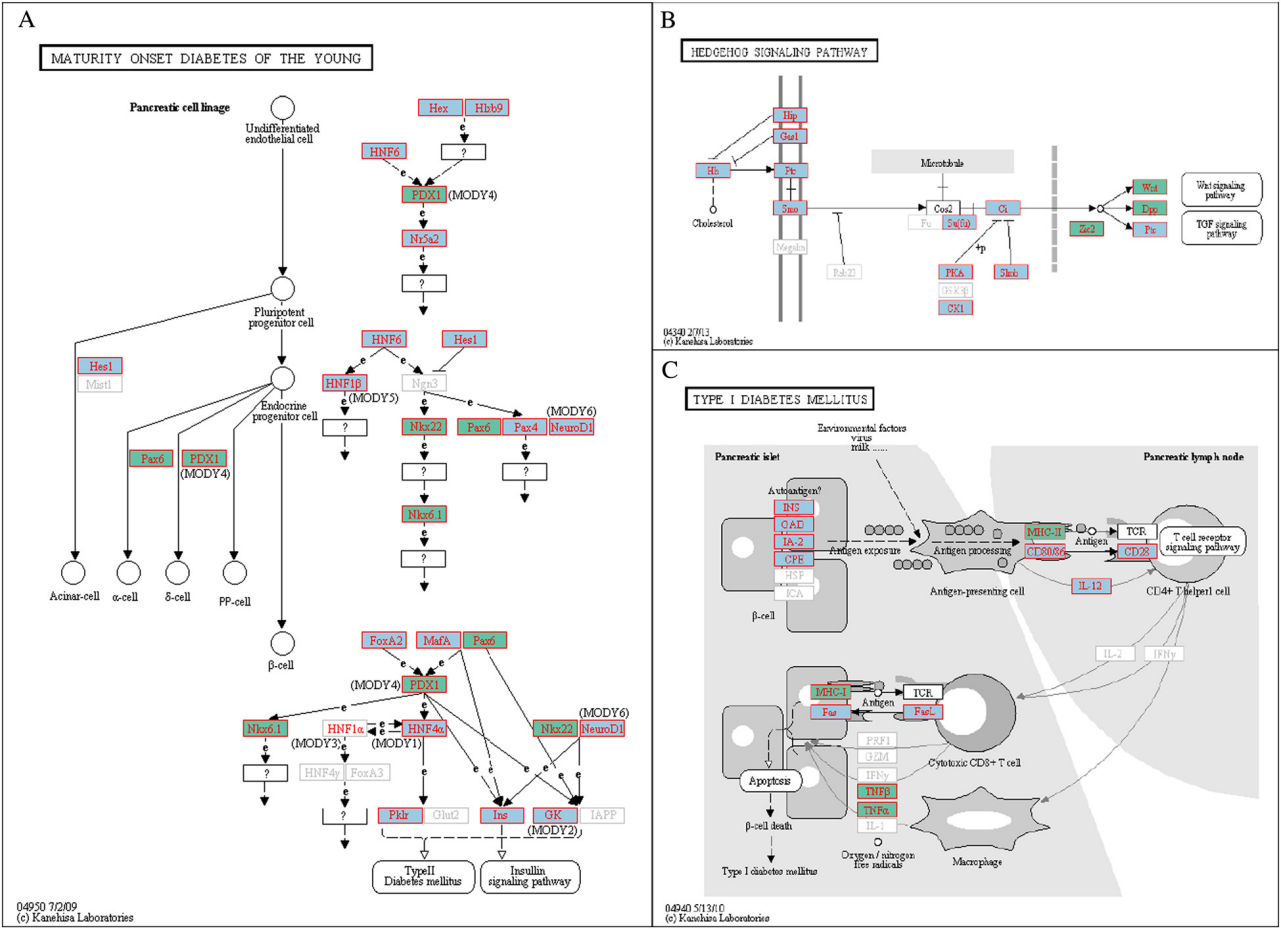
**Top Kegg pathways enriched with aDMCs identified by the FatiGO analysis. (A)** The MODY pathway with identified genes of aDMCs and age-associated DNA methylation sites. Genes found with age-associated sites are shown with a blue background, while genes within an aDMC are shown with a green background. **(B)** The Hedgehog signaling pathway with identified aDMCs and age-associated DNA methylation sites. Genes found with age-associated sites are shown with a blue background, while genes within an aDMC are shown with a green background. **(C)** Type 1 diabetes pathway with identified aDMCs and age-associated DNA methylation sites.

To identify pathways that are enriched in aDMCs associated with age in our cohort in an unidirectional manner, we utilized FatiGO [69] which queried the vast amount of knowledge deposited in the Kyoto Encyclopedia of Genes and Genomes (KEGG) [70,71] and Gene Ontology (GO) databases [72]. We found a number of KEGG pathways and GO terms that are significantly enriched after adjustment in both positively and negatively age-associated clusters. There are a total of 241 genes in these 207 positively age-associated clusters and 16 genes in the 9 negatively associated clusters. As shown in Additional file 8, three KEGG pathways were found to be significantly enriched in clusters positively associated with age, including the hedgehog signaling pathway (adjusted *P* = 3.96 × 10^−3^; Figure 5A) and the maturity-onset diabetes of the young (MODY) pathway (adjusted *P* = 6.26 × 10^−3^; Figure 5B) and neuroactive ligand-receptor interaction (adjusted *P* = 1.58 × 10^−2^). A total of 387 GO terms for biological processes were significantly enriched within the aDMC genes (Additional file 9), as were 55 GO terms for molecular functions (Additional file 10). Type I diabetes mellitus was found to be the most significant pathway enriched in epigenetic clusters negatively associated with age (Figure 5C, Additional file 11). There was no significant enrichment of biological processes and molecular function GO terms within the negative clusters. The biological processes that were significantly enriched in positively associated aDMC genes include development of a multitude of organs and systems from embryonic phases to adulthood, stem cell development and maintenance, cell recognition, motility and migration, regulation of cell differentiation and proliferation and cell cycle, response to temperature and other abiotic stimulus, response to dietary excess, cytokine and insulin secretion, metabolism homeostasis, adult behavior, and aging. The top four GO terms enriched in age-associated epigenetic loci are all related to development of the brain/nervous system. The molecular functions of these genes are also highly enriched in sequence-specific DNA binding.

### Analysis of differential age effects on CpG methylation at known MetS candidate loci in subjects with MetS and those without

**Figure 6:**
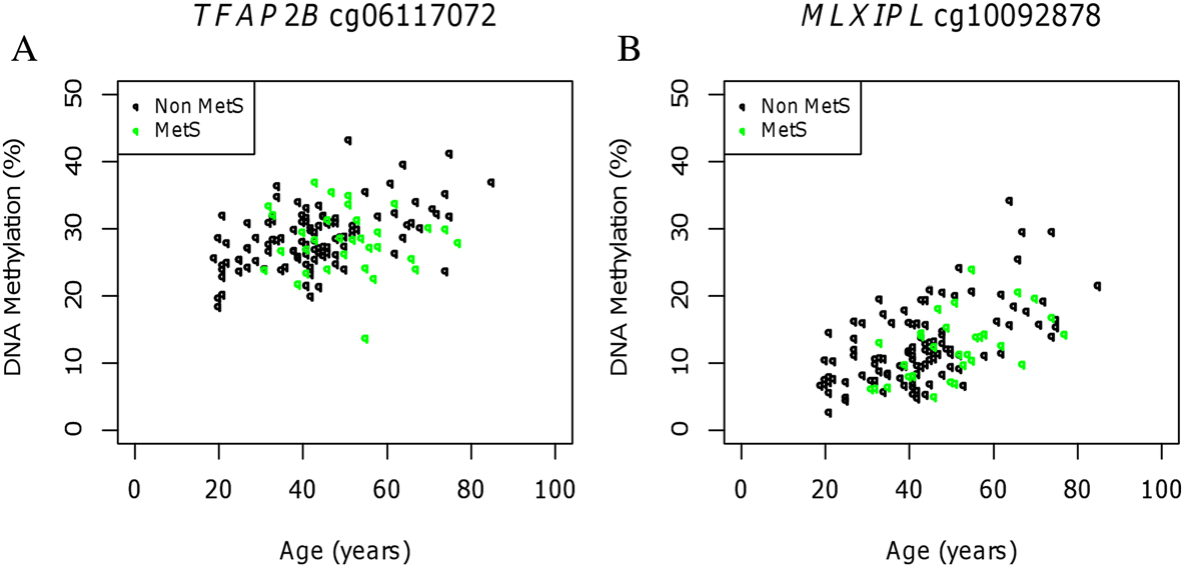
**MetS status and aging rate of candidate CpG site methylation. (A)** Methylation of CpG site cg06117072 located in the gene body of *TFAP2B*, a known MetS gene, with respect to age, in subjects with MetS and those without. **(B)** Methylation of CpG site cg10092878 in the MetS gene *MLXIPL* with respect to age, in subjects with MetS and those without.

We tested the hypothesis that the aging rates for CpG methylation at MetS genes are different in subjects expressing MetS symptoms as compared to subjects without MetS. There are 127 CpG sites which belong to 11 genes previously implicated in MetS genetics (55) in our dataset after QC. Of these, 23 CpG loci were genome-wide significantly associated with age in our cohort. We examined the aging rate of each of these 23 epigenetic markers in the two groups of adult subjects that were separated using the ATPIII definition of MetS (24% with MetS) and found that four sites of four different genes showed nominal differentiation between the two groups (Table 3). Remarkably, we found that not only do the aging rates differ in these two groups by 2.6–5.1 fold but their methylation also changes over time in opposite directions. As shown in Figure 6A, CpG site cg06117072 exhibited increased methylation over age in non-MetS subjects but decreased over time in MetS subjects. In other sites such as cg10092878 in the MetS gene MLX interacting protein-like (*MLXIPL*), the methylation aging rates did not show any significant difference between the two groups of subjects (Figure 6B).

**Table 3:** **MetS status on aging rate in known MetS genes**

**CpG site**	***P***_age_	**Beta (age, all)**	**Beta (age, no MetS)**	**Beta (age, MetS)**	***P***_diff_	**Chr**	**Position (bp)**	**Gene**	**Gene region**
*cg06117072*	1.62 × 10^−15^	0.030	0.027	−0.010	0.004	6	50899344	TFAP2B	Body
*cg04926134*	5.73 × 10^−14^	0.029	0.031	−0.006	0.005	2	165186498	GRB14	First exon
*cg14683125*	1.04 × 10^−16^	0.028	0.023	−0.005	0.023	8	126510565	TRIB1	TSS1500
*cg22697325*	2.64 × 10^−18^	−0.031	−0.020	0.004	0.046	1	39391972	MACF1	Body
cg23485738	5.24 × 10^−09^	−0.023	−0.006	−0.032	0.068	6	32044171	SKIV2L	Body
cg20069688	4.22 × 10^−11^	−0.026	−0.005	0.015	0.134	6	32049028	STK19	Body
cg24641186	5.72 × 10^−23^	0.036	0.027	0.011	0.188	6	50912106	TFAP2B	Body
cg00207280	2.59 × 10^−10^	0.016	0.010	−0.001	0.231	8	126510575	TRIB1	TSS1500
cg07570723	1.53 × 10^−12^	0.024	0.013	0.002	0.338	1	39647825	KIAA0754	5′UTR
cg09247060	3.22 × 10^−27^	0.037	0.024	0.014	0.409	6	50895762	TFAP2B	Body
cg27260772	1.03 × 10^−16^	0.032	0.031	0.020	0.422	6	50899161	TFAP2B	Body
cg24161652	2.25 × 10^−10^	0.026	0.020	0.008	0.433	6	50921818	TFAP2B	3′UTR
cg02244386	4.45 × 10^−08^	−0.020	−0.007	−0.017	0.434	2	165182240	GRB14	Body
cg07737781	2.39 × 10^−22^	0.036	0.031	0.022	0.437	7	72676802	MLXIPL	1stExon
cg19620724	2.11 × 10^−24^	0.038	0.027	0.018	0.461	6	50918811	TFAP2B	Body
cg10092878	6.05 × 10^−25^	0.038	0.031	0.039	0.472	7	72676795	MLXIPL	First exon
cg24366557	2.51 × 10^−32^	0.040	0.034	0.028	0.569	6	50895609	TFAP2B	Body
cg22282405	3.01 × 10^−24^	0.036	0.032	0.027	0.686	6	50918641	TFAP2B	Body
cg07103129	7.92 × 10^−28^	0.040	0.031	0.026	0.703	6	50895923	TFAP2B	Body
cg21317965	5.58 × 10^−12^	0.026	0.019	0.014	0.720	6	50911809	TFAP2B	Body
cg23015341	4.29 × 10^−24^	0.035	0.023	0.021	0.843	6	50921300	TFAP2B	3′UTR
cg13824302	2.01 × 10^−07^	0.021	0.010	0.009	0.929	7	72677002	MLXIPL	TSS200
cg08876103	3.17 × 10^−19^	−0.028	Convergence failure			1	39344910	MACF1	Body

*P*_diff_ is the significance value between the betas in the MetS and non-MetS groups. Italicized CpGs are nominally significant by *P*_diff_.

## Discussion

We report here the first survey of age-associated peripheral blood DNA methylation in a cohort of Northern European origin comprised of large extended families. Using families with extended pedigrees to study the association between age and epigenetic modifications of each interrogated CpG locus can be more powerful than designs using unrelated [34,73], identical and fraternal twins [36,37], or small nuclear pedigrees [74]. It has been shown that genetic effects determine the majority of transgenerational similarity in DNA methylation in humans [23], and by using extended pedigrees, we may have more power to detect age-affected epigenetic signals as compared to designs using unrelated subjects because these families will have less of the epigenetic variation that can be caused by differences in the genetic makeup of unrelated individuals (Additional file 2). By comparing our study with several recent genome-wide age-association studies, we found that the current study has one of the broadest age sample ranges, including children and adolescents, and we were able to identify the majority of previously identified age-associated CpG sites [35,39-41,56,57,75-77] as well as more than 20,000 novel age-associated CpG sites.

Since our study utilized an obesity-prone cohort, it is possible that some of the methylation changes we see in the older subjects are due to the effect of obesity and not due to aging per se. We cannot exclude this possibility, but since we found a large number of loci that have also been discovered in other studies of age-related methylation changes (Additional file 2) that were not related to obesity, we believe that our findings are more likely to be age-related than obesity-related.

We examined the age-associated methylation loci that surpassed genome-wide significance level (after Bonferoni correction, *P*_α = 0.05_ = 3.65 × 10^−7^) in genomic regions densely packed with age-associated CpG sites. We have named these clusters as aDMCs (Additional file 7). We found a total of 290 aDMCs across the autosomal genome with the majority of clusters containing unidirectional, positively associated CpG sites. Only nine clusters were found with CpG sites all negatively associated with age. The sizes of these clusters range from less than 0.5 Kb in a transcription promoter to a nearly 70 Mb region that can span up to four genes.

The families used in the current study all live in rural or semi-urban regions of the states of Wisconsin (six families) and Illinois (one family) and are mostly categorized as working and/or middle class families (TOPS Club Inc., personal communication). There are likely to be broadly similar household routines of dietary intake and activity within these large families. We therefore expect that by using these families to study epigenetic changes over age, we reduce systematic differences in external environmental factors such as geographical location and dietary and lifestyle patterns.

The probands of our TFSE cohort were recruited based on the presence of at least two obese and one never-obese sibling(s) in each family, thus raising the likelihood of having obesity-prone genetic patterns in these families. We hypothesize that since our subjects have an obesity-prone genetic background and live in an obesity-inducing environment, the genomic regions that are strongly associated with age are also enriched in gene groups and pathways involved in metabolism-related cascades.

To find these loci, we first searched in our list of age-associated differentially methylated CpG sites and clusters for any gene(s) previously characterized for a role in the etiology of obesity, T2D, or MetS and inflammation. We found that the majority of previously established obesity, T2D, or MetS and inflammation genes overlap with one or more of our age-associated differentially methylated clusters (Figure 1, Additional files 4, 5, and 6). When we filter these genes based on our clustering criteria, three obesity genes, two T2D genes, and one MetS gene each contains at least one aDMC (Figure 1, Additional files 4, 5, and 6). Some of these genes have been well-characterized previously. For example, the product of gene *IRS2* works as a signaling mediator between cytoplasmic receptor kinases and downstream effectors including *PI3 Kinase*, *Akt*, and *mTOR* [78] and is an important adaptor in cascades regulated by insulin, insulin-like growth factor 1, interleukin 4 (*IL-4*), and other cytokines [79]. Diseases associated with disrupted functions of *IRS2* include fatty liver disease and glucose intolerance, which is a precursor to MetS [80,81].

Single-minded 1 (*SIM1*) is a helix-loop-helix PAS domain transcription factor. Sim, its homologue in Drosophila, is a key factor in determining the differentiation of central nervous system (CNS) midline cells [82]. Losing one functional copy of Sim1 in mice leads to early-onset obesity, hyperinsulinemia, and hyperleptinemia [83]. In humans, disruption of *SIM1* gene locus has been found to have caused profound early-onset obesity [84]. In our analysis, *SIM1* has an aDMC that spans 22,914 bp covering the 5′-untranslated region to exon 8 containing 44 CpG sites that were significantly associated with age including five sites situated in the promoter region (TSS1500 to first exon).

A number of genes known to work in the regulation of food intake and energy balance are found to have one or more CpG markers modifiable by age. These include leptin and related genes *LEP*, *LEPR*, *CNR1*, *DRD2*, and *SLC6A4* as well as the hypothalamic factor *POMC* and its processor *PCSK1* and neurotropic factor *BDNF* and its receptor *NTRK2* [53]. Clinically, disruption of POMC, PCSK1, BDNF, and NTRK2 is associated with severe early-onset obesity [85,86]. *PPARG* is one of the most replicated diabetes and obesity genes, with sequence variants strongly associated with obesity [87] and T2D [88]. We have found two age-associated differentially methylated sites inside the body of the *PPARG* gene.

Multiple obesity and T2D candidate genes identified by the GWAS approach were found to have age-associated DNA methylation associations in our analysis. These genes include extensively replicated genes such as *BDNF*, which is not only shown to be associated with adult obesity but also with childhood obesity [89]. Furthermore, our results include four other genes for childhood obesity (identified by GWAS) that were differentially methylated according to age: *SDCCAG8* and *TNKS*/*MSRA* [90] were first identified in Northern European populations for early-onset extreme obesity whereas *OLFM4* and *HOXB5* are two recently discovered candidate genes for common childhood obesity that were found by combining 14 existing GWAS datasets [91]. Interestingly, we found that the gene *SDCCAG8* contains epigenetic markers that alter their methylation more rapidly in children than in adults (data not shown), suggesting a possible explanation for these genes having more profound effect on clinical phenotypes in children than in later life.

Pathway enrichment analysis reveals that in our family cohort, age-associated DNA methylation sites are densely packed around genes working in pathways such as the hedgehog signaling pathway, the MODY pathway, and T1D pathway (Additional files 8 and 10). These findings suggest intriguing connections between epigenomic profiles and the high risk and high prevalence of obesity and MetS traits in our study subjects. Hedgehog signaling is not only a key regulator of development in both invertebrate and vertebrate systems but it also plays a critical and conserved role in fat formation [92], fat storage, and brown fat and muscle cell metabolism reprograming in animals [93]. Blocking Hh signaling by an antibody in adult mice fed on a high fat diet protects them from gaining weight and developing liver steatosis [94]. The molecular cascade of Hh signaling involves the initial binding of one of the Hh proteins Sonic Hh (*SHH*), Indian Hh (*IHH*), and Desert Hh (*DHH*), with receptor *PACH1* and *PACH2* that release its inhibition on the membrane receptor Smoothened (*SMO*). Released *SMO* then activates a complex signaling cascade, which leads to nuclear translocation of transcription factors of the Gli (*GLI*) family and the resultant activation or repression of downstream genes [95-97]. In our results, age-associated differential methylation was found to be associated with Hh genes *IHH* and *DHH*, Hh receptor genes *PACH1* and *PACH2*, and *SMO* and the downstream effector genes *GLI2* and *GLI3*, and several other regulators of the Hh pathways including *SUFU* and *PKA* (Figure 5). Densely packed age-associated CpG sites, defined as aDMCs, are present in the regulatory region and/or the body of genes of transcription factors *WNT*, *BMPs*, and *Zic2* (Figure 5). Our findings suggest a novel mechanism in which the process of aging influences genomic regulatory marks of a group of genes that work in pathways critical for fat cell fate determination, fat storage, cellular metabolism reprogramming, and diet-dependent regulation of mammalian body weight and lipid metabolism.

Diabetes mellitus, like obesity, is a chronic condition that increases in its prevalence as people age [1,3], but the mechanism underlying this age-dependent risk is not clear. The ‘acquired’ form of diabetes, T2D, is a complex syndrome whose onset is determined by multiple genes and their interactions with the environment [98]. T2D affects an estimated 350 million people in the world today and is caused by complete or partial malfunction in a body’s ability to respond to blood glucose through production of insulin [99,100]. MODY is a type of diabetes that is defined collectively by clinical symptoms caused by mutations in any one of the six genes that work in the MODY pathway. Five of these six genes (*HNF4A*, *HNF1A*, *PDX1*, *HNF1B*, *NeuroD1*) encode transcription factors that regulate β-cell homeostasis and/or insulin production and secretion, and gene *GK* is a glucokinase that is involved in beta cell sensing of blood glucose levels [101]. In our epigenetic study of obesity-prone families, the MODY gene pathway was the second most significant pathway with dense enrichment of DNA methylation CpG sites/clusters strongly associated with age. This finding suggests a novel mechanism in which epigenetic changes affect outcomes of acquired obesity-induced beta-cell and insulin unresponsiveness that leads to T2D. As the patterns in DNA methylation of these loci change over age, it may explain the polygenic, complex, and subtle features that are observed in the development of obesity and T2D and their subsequent course.

In this study, we demonstrated the first evidence of differential methylation aging in MetS genes in MetS subjects as compared with non-MetS subjects. Our results showed an interesting pattern in which the epigenetic changes over age are slower in MetS subjects, and its directionality is the opposite to that in non-MetS subjects in all four of these identified loci (Table 3; Figure 6). Our results suggest that the age effects on epigenetic changes are both genomic locus- and MetS status-dependent. The four nominal MetS-differentiated aging markers are situated at genes that have been previously shown to be metabolism and MetS traits-relevant in genetic or cell biology studies. But the epigenetic implications of these genes in MetS have not been reported. Transcription factor activating enhancer binding protein 2 beta (*TFAP2B*) (cg06117072) encodes a transcription factor from the AP-2 family. This gene has been implicated in dietary weight maintenance [102,103]. Growth factor receptor-bound protein 14 (*GRB14*) (cg04926134) encodes an adaptor protein that binds with insulin receptors and insulin-like growth factor receptors that may have an inhibitory effect on insulin signaling and may play important roles in metabolic homeostasis and growth regulation [104-107]. Tribbles pseudokinase-1 (*TRIB1*) (cg14683125), a mitogen-activated protein kinase (*MAPK*) activation modulator, was found to control migration and proliferation of smooth muscle cells [108] and has been implicated in lipid metabolism [109-111]. Microtubule-actin crosslinking factor 1 (*MACF1*) (cg22697325) is a member of protein family that form bridges between different cytoskeletal elements [112].

With the size of our sample, we do not have enough statistical power to detect all existing signals for MetS-specific aging methylation. We expect that with a larger sample size, one can discover many more disease state-dependent epigenetic markers not only for MetS but also for other aging relevant conditions such as obesity, T2D, dementia, and cancer. This may eventually lead to new clinical approaches in screening and diagnosing people with differential ‘epigenetic risks’ for developing diseases as they age.

We examined the epigenetic changes associated with aging in DNA obtained from peripheral blood, a tissue type that is routinely used as a surrogate for mapping age-related DNA methylation changes [35] because even though DNA methylation (unlike the DNA sequence) is tissue specific, there is a significant portion of the epigenome with patterns of DNA methylation common to multiple tissues [113], and this may be especially true of age-related methylation patterns [40,75]. In addition, a study using peripheral blood has the potential to identify changes in DNA methylation that can be practically used as a diagnostic test in clinic, where blood is much more likely to be available for testing as compared to other tissue samples. However, based on our findings, it will be interesting in a future study to profile some of our age-associated candidate sites in target tissues such as beta cells and to relate these patterns with beta cell function and insulin gene expression.

We conducted our study in a family cohort of Northern European descent. Generalization of our findings requires validation in distinct cohorts with similar pedigree structures. It will also be a valuable expansion of our cross-sectional study if we can recall some of our subjects to obtain longitudinal data on the epigenetic changes as well as on the functional status of metabolic pathways over time in the same individuals. Due to the scope and the focus of the current study, we have not looked at the associations of particular methylation loci with phenotypes in our subjects as this will be approached in the future. The connection between epigenetic status and gene expression in blood and certain target tissue types also warrants further investigation.

Although our mapping utilized one of the array-based epigenetic platforms that gave the highest available genomic site coverage, it has not nearly exhausted the epigenome. A next-generation sequencing-based approach such on methyl-binding domain-isolated genomic sequencing (MiGS), MeDIP-seq, or bisulphite-sequencing [114] will be a way to improve the coverage of all possible epigenetic sites with age.

## Conclusions

We have conducted one of the first genome-wide surveys of age-associated DNA methylation in a family cohort with large extended pedigrees. In families at high risks for developing obesity-related metabolic disorders, we found age-associated genomic loci densely situate near genes that function in the hedgehog signaling pathway and in MODY. These findings suggest a novel mechanism underlying the gradual deleterious effects of multiple genes and their interactions with nutrition over time, which may contribute to obesity and its complications. The results from this study shed light on the relationship between aging and increased prevalence of obesity, T2D, and related abnormalities and thus may lead to novel approaches for early detection and prevention of these health-endangering conditions.

## Methods

### Samples

The study cohort consists of 192 individuals ranging in age from 6 to 85 years old representing seven families. Of these, 53 subjects were 18 years and younger at ascertainment and 106 are females. Details of recruitment and phenotyping procedures have been described previously [47,115]. Briefly, each nuclear family was recruited through an obese proband (BMI ≥ 30) who was a member of TOPS Club with the minimal requirement of the availability of two obese siblings, a least one, preferably both, of the parents and one never-obese (BMI ≤ 27) sib and/or parent. A subsequent extension included the ascertainment of all biologically related members over the age of 18 including aunts, uncles, grandparents, and adult children and their accompanying parent(s). Recently, this cohort was further enhanced by the ascertainment of their children and adolescent descendants (aged 6–18 years) with their accompanying parent(s). Clinical phenotypes for all subjects included weight, height, BMI, waist circumference (WC), hip circumference (HC), waist to hip ratio (WHR), fasting glucose (FG), fasting insulin (FI), insulin to glucose ratio (IGR), homeostasis model assessment (HOMA), plasma triglycerides (TG), total cholesterol (TC), low density lipoprotein cholesterol (LDL-c), high density lipoprotein cholesterol (HDL-c), systolic and diastolic blood pressure (sBP and dBP), and pulse. Total abdominal fat, visceral fat (VF), and subcutaneous fat (SubQF) were measured by computed tomography scans of the fourth lumbar spine [116] in adults and by magnetic resonance imaging at the same level in children and adolescents; circulating levels of adiponectin and leptin were measured by a double antibody equilibrium radioimmunoassay (RIA) (Millipore Corporation, Billerica, MA) and TNF-alpha, interleukin-1beta (IL-1β), and interleukin-6 (IL-6) levels that were measured as previously described [117]. Adult Treatment Panel III (ATPIII) criteria was used to identify adults with MetS. Informed consent was obtained from the participating subjects. All study procedures for adults, adolescents, and children were approved by the Institutional Review Boards of the Medical College of Wisconsin (HRRC#325-94 and HRRC#013-00) and Children’s Hospital of Wisconsin (CHW 04/87), respectively.

### Illumina Methylation 450 k data production

Genomic DNA was isolated from peripheral blood after an overnight fast on the same day when each subject was assayed for obesity and MetS phenotypes; thus, the CpG methylation states profiled from these samples reflect the epigenetic status associated with that individual’s current state of body composition and metabolism. One microgram of human genomic DNA was sodium bisulfite-treated for cytosine (C) to thymine (T) conversion using the EZ DNA Methylation kit (Zymo Research) according to the manufacturer’s guidelines. The converted DNA was purified and prepped for analysis on the Illumina HumanMethylation450 microarray following the manufacturer’s guidelines. The Illumina HumanMethylation450k microarray measures the methylation levels of more than 485,000 methylation sites. It includes CpG sites surrounding the transcription start sites (−200 to −1,500 bp, 5′UTRs and exon 1) for 99% of RefSeq genes, CpG sites within non-coding RNAs, intergenic regions identified in genome-wide association studies as well as CpG islands/shores/shelves and open sea of the genome. CpG annotations (chromosomal location, reference gene, etc.) were identified using the Illumina manifest 1v2.GenomeStudio software and Methylation Module (Illumina) was used to generate final reports containing signal intensities and detection *P* values excluding X and Y chromosomes. No background subtraction or control normalization was applied with GenomeStudio.

### Genomic CpG methylation data QC and processing

For initial quality control preparation of the Infinium Human Methylation 450 K data, we used the Lumi: QN + BMIQ pipeline described previously [118]. Raw signal intensities and detection *P* values of 22 autosomal chromosomes were extracted from GenomeStudio and loaded into Lumi. Next, quality control of the data resulted in the removal of CpG sites with detection *P* value ≥ 0.01 in more than 5% of the samples (471,473 sites left). All samples had at least 99% CpG sites with detection *P* value ≥ 0.01; thus no samples were removed. Recently, multiple groups have reported that this array contains cross-reacting probes that cannot be distinguished between multiple chromosomal positions and that therefore need to be excluded from downstream analysis [119]. Furthermore, studies including ours (Y.Z., unpublished data) have shown that a significant proportion of genomic CpG loci are common polymorphic locations where both C or G or the dinucleotides are changed to a different code, thus abolishing the ability of being methylated in that genome [119]. Considering the inaccuracy these single nucleotide polymorphisms (SNPs) may cause in the quantification of methylation status of these CpG sites, we therefore excluded all known polymorphic CpG sites.

Color bias adjustment (Col.Adj) and quantilenormilzation (QN) were performed on signal intensities as implemented in Lumi. Briefly, the QN works on total signal intensity, assuming that the distributions of the pooled methylated and unmethylated probes are similar for different samples. Intensities were then used to generate Beta values. Within Lumi, ‘*β*’ values are defined as follows:

$$ \beta =\frac{I_{\mathrm{m}}}{I_{\mathrm{U}}+{I}_{\mathrm{m}}+\alpha } $$

where *I*_M_ and *I*_U_ represent the fluorescence intensity originating from methylated or unmethylated CpG locus and *α* is a constant. Beta mixture quantile dilation (BMIQ) was then performed on *β* values of QNed data to account for probe type bias. As the Illumina platforms have been shown to discriminate beta values that differ as little as 17% [120,121], we excluded from analysis probes that ranged <0.17 in *β* values (*n* = 243,711) to ensure probes analyzed might exert a significant biological change. After these steps, a total of 137,168 CpG sites for all 192 samples were imported into data analysis. BMIQ’ed *β* values for probes with ≥0.17 variation were then converted to *M* values for data analysis. Lumi defines *M* values as:

$$ M={ \log}_2\left(\frac{I_{\mathrm{m}}+\alpha }{I_{\mathrm{u}}+\alpha}\right) $$

All analyses were run using *M* values, which are more statistically valid for analysis of differential methylation levels owing to its more homoscedastic nature [122].

### Peripheral blood cell subtype estimation

To estimate cell-type proportions, we used the R minfi package and estimateCellCounts function [123,124]. This method estimated the proportions of six cell types (monocytes, granulocytes, CD8+ T-cells, CD4+ T-cells, NK cells, and B cells) for each individual based on their genome-wide methylation signatures, using an external reference inferred from sample profiles of cell-specific methylation [125]. For each regression test, five of the six proportions were used as covariates.

### Validation by pyrosequencing

DNA methylation at selected sites was validated in a subset of the original cohort by the bisulfite pyrosequencing. This subset consisted of 47 male subjects ages 6 to 21. One microgram of human genomic DNA was sodium bisulfite converted using the EZ DNA Methylation kit (Zymo Research) according to the manufacturer’s guidelines. Pyrosequencing was performed using the PyroMark MD system (Qiagen, Valencia, CA) according to the manufacturer’s protocol. Briefly, the PCR was performed with 10 μM primers, one of which was biotinylated for later purification by Streptavidin Sepharose (VWR). The oligonucleotide primers were purchased from IDT and used for the amplified region of *DDO*: the forward primer, TGTTTAGGAGAAAGGAGTAAGTGATT; the reverse biotinylated primer, ACCCATTATTCACCATACCTACAA; and the pyrosequencing primer, TTTTATGGAGTTGTTTTTGTTAAG. Sepharose beads containing the PCR product were washed and purified using 0.2 M NaOH and the Pyrosequencing Vacuum Prep Tool (QIAGEN). Five microliters of the PCR products was sequenced, and methylation was quantified using the provided software (QIAGEN).

### Statistical analysis

#### Analysis of age-associated CpG loci

The quantitative genetic analyses program SOLAR [52] was used to analyze DNA methylation differences associated with age in the whole cohort. SOLAR is a software package designed to perform tests of genetic and epigenetic association in family data. Parameter estimation by maximum likelihood is performed for both random and fixed effects; in the present context, the random effect of expected allele sharing given pedigree relationships is estimated to account properly for the non-independence of related individuals. For each individual *i*, the value of a trait *Y* is modeled as:

$$ {Y}_i=\mu +{\mathbf{X}}_{\mathbf{i}}\boldsymbol{\upbeta} +{g}_i+{\varepsilon}_i $$

where *μ* is the trait mean, **X**_**i**_ is a vector of fixed effects measured on individual i, **β** is a corresponding vector of regression coefficients, and *g*_i_ and *ε*_i_ are, respectively, a random additive genetic effect and an error term. The covariance of the trait in any two individuals *i*, *j* is decomposed as:

$$ \mathrm{c}\mathrm{o}\mathrm{v}\left({Y}_{\mathrm{i}},\;{Y}_{\mathrm{j}}\right)=2{\phi}_{\mathrm{i},\mathrm{j}}{\upsigma^2}_{\mathrm{g}}\;\mathrm{f}\mathrm{o}\mathrm{r}\;i\ne j $$

$$ \operatorname{cov}\left({Y}_{\mathrm{i}},{Y}_{\mathrm{j}}\right)={\sigma}_{\mathrm{g}}^2+{\sigma}_{\upvarepsilon}^2\mathrm{f}\mathrm{o}\mathrm{r}\;i=j $$

where 2*ϕ*_i,j_ is a kinship coefficient (representing the expected proportion of alleles shared identical by descent for two individuals of a given relationship class) and *σ*^2^_g_ and *σ*^2^_ε_ are, respectively, additive genetic and residual components of variance. Inclusion of the random effect terms appropriately conditions the estimates of the fixed effect parameters on the relatedness of study subjects. Analyses were performed for each CpG site separately, using *M* values, where *M* was modeled as a linear function of age with models that included the random effect of kinship. Sex and cell type composition were included as covariates in all models to account for systematic differences in methylation between men and women. Bonferroni correction for multiple testing, *P*_α=0.05_ = 3.65 × 10^−7^.

#### Analysis of MetS status on aging rate in candidate loci

A candidate CpG site-based regression analysis against MetS status in each subject was performed to determine if there is differential aging of DNA methylation in subjects with metabolic syndrome compared to those without. In this model, two tests were done: in one, the slopes and intercepts of the regression lines are allowed to differ by MetS status, and in another, a null is forced to be the same. The test of statistically equal intercepts asks whether methylation differs by MetS status for the measured span of ages. The test of equal slopes asks whether MetS impacts change in methylation with age.

#### Identification of genomic clusters of age-associated CpGs

The R package bump hunter [67] was used to identify genomic clusters of age-associated CpG sites. The clusterMaker function within the bump hunter package was applied to the genome-wide age-associated CpGs, and clusters are formed if two positions are within 10 kb of each other. Each chromosome is clustered independently from each other.

To account for array bias, we took the minimum and maximum position of each cluster and looked at the total number of probes from that region that were originally implemented into the analysis. To further define our aDMCs, at least ten CpG sites had to be in the original data set which was implemented into the analysis. At least 50% of those sites had to be genome-wide significantly associated with age. Through this method, we identified a total of 246 aDMCs throughout the autosomal genome. We further looked at the direction of effect age has on each CpG within the identified clusters. If 100% of the CpGs in the cluster had the same direction of effect, it was labeled as ‘positive’ or ‘negative’. If there was variable direction of effects within the cluster, it was labeled as ‘varying’.

### Gene ontologies and pathway analysis of aDMCs

Gene Ontology analysis was done with the FatiGO tool [69], which uses Fisher’s exact test to detect significant overrepresentation of GO terms and disease pathways. FatiGO pools multiple databases, such as the Gene Ontology (GO) terms [72] and Kyoto Encyclopedia of Genes and Genomes (KEGG) [70,71]. In our study, the set of one direction cluster genes were analyzed for overrepresentation against the rest of the genome. Multiple test correction to account for the multiple hypothesis tested (one for each term) is applied to reduce false positives. GO terms and KEGG pathways with adjusted *P* value < 0.05 are considered significant.

## Additional files

Additional file 1:
**Data cleaning pipeline for probed Illumina 450 k methylation signals.**
Additional file 2:
**Summary of age-associated DNA methylation signals identified in the current and previous studies.**
Additional file 3:
**Comparison of age-associated CpG sites identified in this study with sites identified in previous studies.**
Additional file 4:
**Previously known obesity genes with CpG sites found to be age associated in TFSE.**
Additional file 5:
**Previously known type 2 diabetes genes with CpG sites found to be age associated in TFSE.**
Additional file 6:
**Previously known MetS genes with CpG sites found to be age associated in TFSE.**
Additional file 7:
**Autosomal map of age-associated differentially methylated CpG clusters (aDMCs).**
Additional file 8:
**KEGG pathways significantly enriched in positive aDMCs identified by the FatiGO analysis.**
Additional file 9:
**GO Biological Processes significantly enriched in positive aDMCs.**
Additional file 10:
**GO Molecular Functions significantly enriched in positive aDMCs.**
Additional file 11:
**KEGG pathways significantly enriched in negative aDMCs identified by the FatiGO analysis.**
