# Supplementary material for: An epigenetic map of age-associated autosomal loci in northern European families at high risk for the metabolic syndrome
Source: Clin Epigenetics. 2015 Feb 20;7(1):12. doi: 10.1186/s13148-015-0048-6 (PMC4372177; doi:10.1186/s13148-015-0048-6)
Supplement: Additional file 1: — Data cleaning pipeline for probed Illumina 450 k methylation signals. [file 13148_2015_48_MOESM1_ESM.docx]

**Additional File 1.** Quality control filtering pipeline for Illumina 450k methylation probes.

**
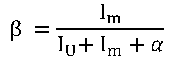
**

**Sample and probe filtering**

detection P-value ≥ 0.01 in more than 5% of the samples n=2123

Filtering of CpGs near or on SNPs or cross reactive probes (Chen et al. 2013), n = 90865

**Color-bias adjustment**

**Quantile Normalization**

**Beta Value Calculations**

**BMIQ Normalization**

**Filtering of probes with <0.17 variation**

n= 243711

Data analysis

n=137168

**M Value Calculations**

**GenomeStudio**

reports containing signal intensities and detection p-values, no background subtraction or control normalization excluding X and Y chromosomes, n=473,867

n=137168

n= 380879

To account for Infinium I or Infinium II probe bias

n=137168

n= 380879

n= 380879

n= 380879

n= 380879
