# Supplementary material for: An epigenetic map of age-associated autosomal loci in northern European families at high risk for the metabolic syndrome
Source: Clin Epigenetics. 2015 Feb 20;7(1):12. doi: 10.1186/s13148-015-0048-6 (PMC4372177; doi:10.1186/s13148-015-0048-6)
Supplement: Additional file 2: — Summary of age-associated DNA methylation signals identified in the current and previous studies. [file 13148_2015_48_MOESM2_ESM.docx]

**Additional File 2. Summary of age-associated DNA methylation signals identified in the current and previous studies**

| Reference | Sample N | Age range (yrs) | Tissue | Array | Number of sites associated with age | Number of available sites in our panel after data cleaning | Shared age associated sites (p<3.65 x 10^-7^) | Significance Cutoff |
| --- | --- | --- | --- | --- | --- | --- | --- | --- |
| Hannum [39] | 656 | 19-101 | Blood | Illumina 450k | 89 | 69 | 69 (100%) | Customized modeling in addition to  FDR <0.05 |
| Day [40] | 71 | 55 ± 14.5 | Blood | Illumina 27k | 961 | 506 | 395  (78%) | FDR <0.05 |
| Teschendorff [41] | 261 | 50-85 | Blood | Illumina 27k | 589 | 375 | 208 (55%) | FDR<0.05 |
|  | 188 | 24-74 | Blood (Type 1 Diabetes) | Illumina 27k | 683 | 453 | 345 (76%) | FDR<0.05 |
| Alisch [35] | 398 | 3-17 | Blood | Illumina 27k | 2078 | 1273 | 488 (38%) | FDR<0.01 |
|  | 78 | 1-16 | Blood | Illumina 450k | 41895 | Data not available |  | FDR<0.01 |
| Garagnani [56] | 64 | 9-83 | Blood | Illumina 450k | 9 | 8 | 8 (100%) | p<2.2 x 10^-8^ |
| Florath [57] | 498 | 50-75 | Blood | Illumina 450k | 162 | 119 | 119 (100%) | p<2.5 x 10^-4^  (top 200 sites) |
| Bocklandt [77] | 34 | 21-55 | Saliva | Illumina 27k | 88 | 65 | 55 (85%) | q<0.05 |
| Horvath [75] | 2434 | 1-101 | Brain and Blood | Both | 1000 | 448 | 400 (89%) | Top 1000 sites based on kME |
| Horvath [76] | 8000 | 0-100 | 51 healthy tissues | Both | 353 | 151 | 77 (51%) | Elastic net regression model |
| **Current Study** | 192 | 6-85 | Blood | Illumina 450k | 22122 | NA | NA | p<3.65 x 10^-7^ |
