# Supplementary material for: An epigenetic map of age-associated autosomal loci in northern European families at high risk for the metabolic syndrome
Source: Clin Epigenetics. 2015 Feb 20;7(1):12. doi: 10.1186/s13148-015-0048-6 (PMC4372177; doi:10.1186/s13148-015-0048-6)
Supplement: Additional file 4: — Previously known obesity genes with CpG sites found to be age associated in TFSE. [file 13148_2015_48_MOESM4_ESM.docx]

**Additional File 4. Previously known obesity genes with CpG sites found to be age associated in TFSE.**

| **CpG Site** | **Page** | **Regression Coefficient** | **Chr** | **Position** | **Gene** | **Region** |
| --- | --- | --- | --- | --- | --- | --- |
| cg26876444 | 6.75E-14 | 0.02 | 1 | 65660001 | LEPR | 5'UTR |
| cg00630958 | 1.74E-19 | 0.03 | 1 | 65764049 | LEPR | 5'UTR |
| cg16987305 | 3.40E-17 | 0.03 | 1 | 65764184 | LEPR | 5'UTR |
| cg03514351 | 6.95E-09 | 0.02 | 1 | 65764353 | LEPR | 5'UTR |
| cg00962740 | 3.41E-11 | -0.03 | 1 | 241574155 | SDCCAG8 | Body |
| cg06882058 | 1.21E-10 | 0.02 | 1 | 241713410 | SDCCAG8 | Body |
| cg23166773 | 5.00E-18 | -0.02 | 1 | 241725639 | SDCCAG8 | Body |
| cg13025668 | 6.49E-13 | 0.03 | 2 | 25245160 | POMC | TSS200 |
| cg20387815 | 1.03E-13 | 0.03 | 2 | 25245170 | POMC | TSS200 |
| cg24425171 | 4.62E-10 | 0.02 | 2 | 25245215 | POMC | TSS200 |
| cg04908300 | 1.52E-08 | 0.02 | 3 | 12305532 | PPARG | 5'UTR |
| cg10499651 | 1.38E-11 | -0.02 | 3 | 12440415 | PPARG | Body |
| cg05044994 | 1.96E-10 | 0.02 | 3 | 189379299 | LPP | 5'UTR |
| cg04773529 | 3.15E-20 | 0.03 | 3 | 189708236 | LPP | Body |
| cg19456953 | 5.94E-21 | 0.04 | 5 | 95794174 | PCSK1 | Body |
| cg09786257 | 2.96E-14 | 0.03 | 5 | 95794451 | PCSK1 | 1stExon |
| cg00637687 | 6.24E-13 | 0.03 | 5 | 95794671 | PCSK1 | 1stExon |
| cg17803175 | 5.35E-13 | 0.03 | 5 | 95794827 | PCSK1 | TSS200 |
| cg02115960 | 2.87E-08 | 0.02 | 5 | 95794835 | PCSK1 | TSS200 |
| cg09879107 | 3.97E-12 | -0.03 | 6 | 88918909 | CNR1 | 5'UTR |
| cg04859726 | 5.58E-25 | 0.04 | 6 | 101001234 | SIM1 | Body |
| cg18782604 | 3.02E-16 | 0.03 | 6 | 101001771 | SIM1 | Body |
| cg06386307 | 1.55E-21 | 0.04 | 6 | 101001932 | SIM1 | Body |
| cg14717557 | 6.72E-16 | 0.03 | 6 | 101002339 | SIM1 | Body |
| cg08979319 | 5.60E-22 | 0.03 | 6 | 101002776 | SIM1 | Body |
| cg16565394 | 4.75E-11 | 0.03 | 6 | 101003395 | SIM1 | Body |
| cg05100070 | 4.50E-14 | 0.03 | 6 | 101003481 | SIM1 | Body |
| cg01978237 | 8.06E-12 | 0.03 | 6 | 101003524 | SIM1 | Body |
| cg03830329 | 1.17E-11 | 0.03 | 6 | 101003802 | SIM1 | Body |
| cg26099134 | 2.13E-15 | 0.03 | 6 | 101004030 | SIM1 | Body |
| cg14314744 | 3.96E-20 | 0.04 | 6 | 101004166 | SIM1 | Body |
| cg13365524 | 2.65E-21 | 0.04 | 6 | 101008814 | SIM1 | Body |
| cg26954968 | 3.24E-11 | 0.03 | 6 | 101010282 | SIM1 | Body |
| cg24211504 | 3.16E-20 | 0.03 | 6 | 101010296 | SIM1 | Body |
| cg00434010 | 4.28E-27 | 0.04 | 6 | 101010412 | SIM1 | Body |
| cg03290400 | 1.25E-10 | 0.03 | 6 | 101010630 | SIM1 | Body |
| cg00736459 | 7.19E-08 | 0.02 | 6 | 101011097 | SIM1 | Body |
| cg23588121 | 8.29E-12 | 0.03 | 6 | 101011759 | SIM1 | Body |
| cg07973435 | 9.03E-26 | 0.04 | 6 | 101012179 | SIM1 | Body |
| cg04337653 | 3.83E-12 | 0.03 | 6 | 101012308 | SIM1 | Body |
| cg26796341 | 2.67E-14 | 0.03 | 6 | 101012412 | SIM1 | Body |
| cg01912955 | 2.63E-09 | 0.02 | 6 | 101013104 | SIM1 | Body |
| cg10745499 | 9.15E-14 | 0.03 | 6 | 101013406 | SIM1 | Body |
| cg06708634 | 7.01E-15 | 0.03 | 6 | 101013616 | SIM1 | Body |
| cg12865837 | 1.98E-08 | 0.02 | 6 | 101018247 | SIM1 | 1stExon |
| cg11891393 | 5.41E-10 | 0.03 | 6 | 101018448 | SIM1 | TSS200 |
| cg22478310 | 8.64E-14 | 0.03 | 6 | 101018465 | SIM1 | TSS200 |
| cg27252696 | 1.36E-25 | 0.04 | 6 | 101019661 | SIM1 | TSS1500 |
| cg12233363 | 2.31E-13 | 0.03 | 6 | 101019751 | SIM1 | TSS1500 |
| cg16038868 | 8.66E-08 | -0.02 | 8 | 10230029 | MSRA | Body |
| cg15633056 | 6.47E-11 | -0.02 | 8 | 10274292 | MSRA | Body |
| cg13723118 | 1.98E-16 | 0.03 | 9 | 86474542 | NTRK2 | 5'UTR |
| cg03628748 | 3.23E-15 | 0.03 | 9 | 86474953 | NTRK2 | 5'UTR |
| cg08470639 | 6.92E-23 | 0.04 | 9 | 86475006 | NTRK2 | 5'UTR |
| cg13698224 | 3.45E-07 | -0.02 | 9 | 86499214 | NTRK2 | Body |
| cg13620631 | 1.60E-10 | -0.02 | 9 | 86679348 | NTRK2 | 3'UTR |
| cg25835733 | 2.85E-08 | 0.02 | 10 | 26543903 | GAD2 | TSS1500 |
| cg07440775 | 1.40E-08 | 0.02 | 10 | 26547389 | GAD2 | Body |
| cg09022607 | 9.41E-08 | 0.02 | 10 | 114702685 | TCF7L2 | Body |
| cg27162705 | 9.19E-08 | 0.02 | 10 | 114703014 | TCF7L2 | Body |
| cg11748187 | 5.39E-08 | 0.02 | 10 | 114703098 | TCF7L2 | Body |
| cg00159523 | 2.92E-07 | 0.02 | 10 | 114703177 | TCF7L2 | Body |
| cg27062243 | 3.60E-07 | -0.02 | 10 | 114823710 | TCF7L2 | Body |
| cg03510732 | 3.27E-07 | -0.02 | 10 | 114861921 | TCF7L2 | Body |
| cg23947039 | 3.33E-09 | 0.02 | 11 | 27678613 | BDNF | Body |
| cg17413943 | 5.08E-08 | 0.02 | 11 | 27696403 | BDNF | Body |
| cg13974632 | 3.18E-36 | 0.04 | 11 | 27697389 | BDNF | Body |
| cg05733135 | 1.40E-27 | 0.04 | 11 | 27697452 | BDNF | Body |
| cg22043168 | 2.98E-27 | 0.04 | 11 | 27697653 | BDNF | Body |
| cg26949694 | 2.61E-25 | 0.04 | 11 | 27698636 | BDNF | Body |
| cg11718030 | 3.30E-13 | 0.03 | 11 | 27700939 | BDNF | TSS1500 |
| cg24249411 | 1.09E-11 | 0.03 | 11 | 27701335 | BDNF | TSS1500 |
| cg18248586 | 2.79E-09 | -0.02 | 11 | 112834236 | DRD2 | 5'UTR |
| cg22458194 | 6.00E-24 | 0.04 | 11 | 112850896 | DRD2 | 5'UTR |
| cg23881278 | 9.29E-16 | 0.03 | 11 | 112851509 | DRD2 | TSS1500 |
| cg12758687 | 1.83E-26 | 0.04 | 11 | 112851537 | DRD2 | TSS1500 |
| cg20629239 | 5.45E-10 | 0.02 | 11 | 112851701 | DRD2 | TSS1500 |
| cg08119452 | 2.26E-07 | -0.02 | 13 | 52500386 | OLFM4 | TSS1500 |
| cg16766249 | 1.41E-09 | 0.02 | 14 | 78814907 | NRXN3 | Body |
| cg14335579 | 3.24E-11 | 0.03 | 14 | 78815750 | NRXN3 | Body |
| cg04272011 | 4.30E-11 | 0.02 | 14 | 78816112 | NRXN3 | Body |
| cg05175318 | 1.29E-28 | 0.04 | 14 | 78817527 | NRXN3 | Body |
| cg24020215 | 1.32E-18 | 0.03 | 14 | 78818394 | NRXN3 | Body |
| cg20076659 | 3.22E-14 | 0.03 | 14 | 78829800 | NRXN3 | Body |
| cg08186935 | 2.49E-11 | 0.03 | 14 | 78914321 | NRXN3 | Body |
| cg07177395 | 3.23E-11 | 0.03 | 14 | 79038439 | NRXN3 | Body |
| cg20055861 | 1.59E-10 | 0.01 | 15 | 65842347 | MAP2K5 | Body |
| cg05951817 | 1.43E-08 | 0.02 | 17 | 25586268 | SLC6A4 | 5'UTR |
| cg22584138 | 1.85E-07 | 0.02 | 17 | 25586346 | SLC6A4 | 5'UTR |
| cg03363743 | 1.45E-23 | 0.03 | 17 | 25586600 | SLC6A4 | 5'UTR |
| cg14692377 | 1.53E-37 | 0.04 | 17 | 25586811 | SLC6A4 | 1stExon |
| cg00690402 | 2.10E-15 | 0.03 | 17 | 44024565 | LOC404266 | TSS200 |
| cg04689379 | 9.33E-11 | -0.03 | 19 | 50863102 | GIPR | TSS1500 |
| cg00848780 | 1.19E-33 | 0.04 | 19 | 50866295 | GIPR | Body |
| cg20901882 | 7.92E-23 | 0.04 | 19 | 50866317 | GIPR | Body |
| cg08264859 | 6.25E-11 | 0.03 | 19 | 50866405 | GIPR | Body |
| cg16082401 | 9.50E-25 | 0.04 | 19 | 50873061 | GIPR | Body |
| cg20434926 | 1.49E-20 | 0.03 | 19 | 50873114 | GIPR | Body |
| cg18735402 | 2.91E-15 | 0.03 | 19 | 50873170 | GIPR | Body |
|  |  |  |  |  |  |  |
