# Supplementary material for: An epigenetic map of age-associated autosomal loci in northern European families at high risk for the metabolic syndrome
Source: Clin Epigenetics. 2015 Feb 20;7(1):12. doi: 10.1186/s13148-015-0048-6 (PMC4372177; doi:10.1186/s13148-015-0048-6)
Supplement: Additional file 5: — Previously known type 2 diabetes genes with CpG sites found to be age associated in TFSE. [file 13148_2015_48_MOESM5_ESM.docx]

**Additional File 5. Previously known Type 2 Diabetes genes with CpG sites found to be age associated in TFSE.**

| **CpG Site** | **Page** | **Regression Coefficient** | **Chromosome** | **Position** | **Gene** | **Region** |
| --- | --- | --- | --- | --- | --- | --- |
| cg23166773 | 5E-18 | -0.02 | 1 | 241725639 | SDCCAG8 | Body |
| cg24739596 | 1.37E-09 | 0.02 | 1 | 242034004 | AKT3 | Body |
| cg11314684 | 2.47E-12 | -0.02 | 1 | 242072911 | AKT3 | Body |
| cg01649611 | 1.27E-21 | -0.02 | 2 | 43374570 | THADA | Body |
| cg06714480 | 2.11E-10 | 0.03 | 2 | 182252552 | NEUROD1 | 5'UTR |
| cg00935043 | 7.48E-08 | -0.02 | 2 | 227306847 | IRS1 | 3'UTR |
| cg08166272 | 4.75E-12 | 0.03 | 2 | 227371395 | IRS1 | 1stExon |
| cg04908300 | 1.52E-08 | 0.02 | 3 | 12305532 | PPARG | 5'UTR |
| cg10499651 | 1.38E-11 | -0.02 | 3 | 12440415 | PPARG | Body |
| cg22177868 | 2.95E-09 | 0.02 | 3 | 64642770 | ADAMTS9 | Body |
| cg22081832 | 3.74E-12 | 0.03 | 3 | 64645499 | ADAMTS9 | Body |
| cg07891473 | 1.64E-15 | 0.03 | 3 | 64645680 | ADAMTS9 | Body |
| cg21938436 | 2.67E-16 | 0.03 | 3 | 64646025 | ADAMTS9 | Body |
| cg11427510 | 1.89E-13 | 0.03 | 3 | 64648535 | ADAMTS9 | TSS200 |
| cg08797864 | 4.49E-10 | -0.02 | 3 | 187013952 | IGF2BP2 | Body |
| cg05044994 | 1.96E-10 | 0.02 | 3 | 189379299 | LPP | 5'UTR |
| cg04773529 | 3.15E-20 | 0.03 | 3 | 189708236 | LPP | Body |
| cg04418434 | 1.67E-15 | 0.03 | 6 | 7055772 | RREB1 | 5'UTR |
| cg24899545 | 6.49E-10 | 0.02 | 6 | 7057845 | RREB1 | 5'UTR |
| cg02010481 | 2.05E-21 | -0.02 | 7 | 28185049 | JAZF1 | Body |
| cg11194698 | 3.01E-10 | 0.02 | 7 | 44151365 | GCK | Body |
| cg14871932 | 1.38E-18 | 0.03 | 7 | 44151848 | GCK | Body |
| cg23381646 | 1.92E-10 | 0.02 | 7 | 44153835 | GCK | Body |
| cg17959631 | 5.22E-12 | -0.02 | 7 | 44156209 | GCK | Body |
| cg03639919 | 2.33E-08 | -0.02 | 7 | 44157836 | GCK | Body |
| cg14689537 | 3.38E-15 | 0.03 | 10 | 94440846 | HHEX | Body |
| cg24787755 | 7.7E-14 | 0.02 | 10 | 94440996 | HHEX | Body |
| cg03330490 | 1.95E-08 | 0.02 | 10 | 94442216 | HHEX | Body |
| cg00876273 | 1.33E-08 | 0.02 | 10 | 94442291 | HHEX | Body |
| cg09022607 | 9.41E-08 | 0.02 | 10 | 114702685 | TCF7L2 | Body |
| cg27162705 | 9.19E-08 | 0.02 | 10 | 114703014 | TCF7L2 | Body |
| cg11748187 | 5.39E-08 | 0.02 | 10 | 114703098 | TCF7L2 | Body |
| cg00159523 | 2.92E-07 | 0.02 | 10 | 114703177 | TCF7L2 | Body |
| cg27062243 | 0.00000036 | -0.02 | 10 | 114823710 | TCF7L2 | Body |
| cg03510732 | 3.27E-07 | -0.02 | 10 | 114861921 | TCF7L2 | Body |
| cg23267890 | 6.98E-12 | 0.03 | 11 | 2421546 | KCNQ1 | TSS1500 |
| cg11025829 | 6.01E-10 | 0.02 | 11 | 2421712 | KCNQ1 | TSS1500 |
| cg24079038 | 2E-09 | 0.02 | 11 | 2421855 | KCNQ1 | TSS1500 |
| cg04104132 | 8.29E-33 | 0.04 | 11 | 2422016 | KCNQ1 | TSS1500 |
| cg19779211 | 1.72E-13 | 0.03 | 11 | 2422067 | KCNQ1 | TSS1500 |
| cg27364242 | 4.79E-08 | -0.02 | 11 | 2427640 | KCNQ1 | Body |
| cg24376802 | 6.77E-09 | -0.02 | 11 | 2441626 | KCNQ1 | Body |
| cg19670883 | 6.8E-09 | 0.02 | 11 | 2458933 | KCNQ1 | Body |
| cg12949760 | 7.09E-08 | -0.01 | 11 | 2499438 | KCNQ1 | Body |
| cg27491887 | 1.73E-27 | 0.04 | 11 | 2511159 | KCNQ1 | Body |
| cg18729298 | 6.77E-28 | 0.03 | 11 | 2511779 | KCNQ1 | Body |
| cg25175863 | 3.46E-09 | -0.02 | 11 | 2560967 | KCNQ1 | Body |
| cg16811455 | 1.56E-07 | -0.02 | 11 | 2699129 | KCNQ1 | Body |
| cg04894537 | 2.17E-08 | -0.01 | 11 | 2719747 | KCNQ1 | Body |
| cg01917852 | 2.09E-07 | -0.02 | 11 | 2729335 | KCNQ1 | Body |
| cg16434897 | 2.57E-07 | -0.02 | 11 | 2729375 | KCNQ1 | Body |
| cg05438727 | 8.24E-18 | 0.02 | 11 | 2756623 | KCNQ1 | Body |
| cg13859639 | 2.58E-09 | -0.01 | 11 | 2803292 | KCNQ1 | Body |
| cg17333973 | 1.9E-13 | -0.02 | 11 | 2803595 | KCNQ1 | Body |
| cg12141659 | 1.65E-12 | 0.02 | 11 | 2804038 | KCNQ1 | Body |
| cg10151367 | 2.55E-20 | 0.03 | 11 | 2804218 | KCNQ1 | Body |
| cg03181582 | 2.51E-19 | 0.03 | 11 | 17455106 | ABCC8 | TSS200 |
| cg18097532 | 6.47E-19 | 0.03 | 11 | 17455117 | ABCC8 | TSS200 |
| cg05803237 | 1.82E-09 | 0.02 | 11 | 92342276 | MTNR1B | TSS200 |
| cg15842276 | 4.18E-09 | 0.02 | 11 | 92342296 | MTNR1B | TSS200 |
| cg01722932 | 6.12E-08 | 0.02 | 11 | 92342301 | MTNR1B | TSS200 |
| cg23049823 | 9.98E-10 | -0.02 | 11 | 92355287 | MTNR1B | 3'UTR |
| cg04408488 | 1.35E-11 | 0.03 | 13 | 27392116 | PDX1 | TSS200 |
| cg04633225 | 1.9E-08 | 0.02 | 13 | 27392161 | PDX1 | TSS200 |
| cg03907847 | 3.81E-09 | 0.02 | 13 | 27393908 | PDX1 | Body |
| cg18064631 | 1.87E-12 | 0.03 | 13 | 27394413 | PDX1 | Body |
| cg03860037 | 2.43E-10 | 0.03 | 13 | 27395359 | PDX1 | Body |
| cg24601716 | 3.52E-28 | 0.04 | 13 | 27396451 | PDX1 | Body |
| cg26299169 | 2.65E-12 | 0.03 | 13 | 27396956 | PDX1 | 3'UTR |
| cg00184376 | 3.38E-30 | 0.04 | 13 | 27397045 | PDX1 | 3'UTR |
| cg05404236 | 2.27E-32 | 0.04 | 13 | 109235094 | IRS2 | 1stExon |
| cg10488031 | 1.7E-20 | 0.03 | 13 | 109235562 | IRS2 | 1stExon |
| cg16851425 | 2.95E-23 | 0.04 | 13 | 109235760 | IRS2 | 1stExon |
| cg06293108 | 2.36E-19 | 0.03 | 13 | 109236257 | IRS2 | 1stExon |
| cg08510264 | 2.02E-14 | 0.03 | 13 | 109236289 | IRS2 | 1stExon |
| cg02320481 | 1.15E-17 | 0.03 | 13 | 109236579 | IRS2 | 5'UTR |
| cg08057103 | 1.66E-12 | 0.03 | 13 | 109236917 | IRS2 | TSS200 |
| cg25802424 | 2.82E-07 | 0.02 | 13 | 109236938 | IRS2 | TSS200 |
| cg08625260 | 1.59E-12 | 0.03 | 13 | 109237235 | IRS2 | TSS1500 |
| cg14694075 | 1.67E-09 | -0.02 | 17 | 33133943 | HNF1B | Body |
| cg05110178 | 1.61E-10 | 0.03 | 17 | 33178066 | HNF1B | Body |
| cg19378036 | 1.99E-07 | 0.02 | 17 | 33179477 | HNF1B | TSS1500 |
| cg23834593 | 9.29E-09 | -0.02 | 20 | 42417334 | HNF4A | TSS1500 |
| cg16121136 | 1.12E-12 | -0.03 | 20 | 42417623 | HNF4A | TSS1500 |
