# Supplementary material for: An epigenetic map of age-associated autosomal loci in northern European families at high risk for the metabolic syndrome
Source: Clin Epigenetics. 2015 Feb 20;7(1):12. doi: 10.1186/s13148-015-0048-6 (PMC4372177; doi:10.1186/s13148-015-0048-6)
Supplement: Additional file 6: — Previously known MetS genes with CpG sites found to be age associated in TFSE. [file 13148_2015_48_MOESM6_ESM.docx]

**Additional File 6. Previously known MetS genes with CpG sites found to be age associated in TFSE.**

| CpG Site | P_age_ | Regression Coefficient | Chromosome | Position | Gene | Region |
| --- | --- | --- | --- | --- | --- | --- |
| cg00207280 | 2.59E-10 | 0.02 | 8 | 126510575 | TRIB1 | TSS1500 |
| cg02244386 | 4.45E-08 | -0.02 | 2 | 165182240 | GRB14 | Body |
| cg04926134 | 5.73E-14 | 0.03 | 2 | 165186498 | GRB14 | 1stExon |
| cg06117072 | 1.62E-15 | 0.03 | 6 | 50899344 | TFAP2B | Body |
| cg07103129 | 7.92E-28 | 0.04 | 6 | 50895923 | TFAP2B | Body |
| cg07570723 | 1.53E-12 | 0.02 | 1 | 39647825 | KIAA0754 | 5'UTR |
| cg07737781 | 2.39E-22 | 0.04 | 7 | 72676802 | MLXIPL | 1stExon |
| cg08876103 | 3.17E-19 | -0.03 | 1 | 39344910 | MACF1 | Body |
| cg09247060 | 3.22E-27 | 0.04 | 6 | 50895762 | TFAP2B | Body |
| cg10092878 | 6.05E-25 | 0.04 | 7 | 72676795 | MLXIPL | 1stExon |
| cg13824302 | 2.01E-07 | 0.02 | 7 | 72677002 | MLXIPL | TSS200 |
| cg14683125 | 1.04E-16 | 0.03 | 8 | 126510565 | TRIB1 | TSS1500 |
| cg19620724 | 2.11E-24 | 0.04 | 6 | 50918811 | TFAP2B | Body |
| cg20069688 | 4.22E-11 | -0.03 | 6 | 32049028 | STK19 | Body |
| cg21317965 | 5.58E-12 | 0.03 | 6 | 50911809 | TFAP2B | Body |
| cg22282405 | 3.01E-24 | 0.04 | 6 | 50918641 | TFAP2B | Body |
| cg22697325 | 2.64E-18 | -0.03 | 1 | 39391972 | MACF1 | Body |
| cg23015341 | 4.29E-24 | 0.04 | 6 | 50921300 | TFAP2B | 3'UTR |
| cg23485738 | 5.24E-09 | -0.02 | 6 | 32044171 | SKIV2L | Body |
| cg24161652 | 2.25E-10 | 0.03 | 6 | 50921818 | TFAP2B | 3'UTR |
| cg24366557 | 2.51E-32 | 0.04 | 6 | 50895609 | TFAP2B | Body |
| cg24641186 | 5.72E-23 | 0.04 | 6 | 50912106 | TFAP2B | Body |
| cg27260772 | 1.03E-16 | 0.03 | 6 | 50899161 | TFAP2B | Body |
