# Supplementary figures and images for: An epigenetic map of age-associated autosomal loci in northern European families at high risk for the metabolic syndrome
Source: Clin Epigenetics. 2015 Feb 20;7(1):12. doi: 10.1186/s13148-015-0048-6 (PMC4372177; doi:10.1186/s13148-015-0048-6)

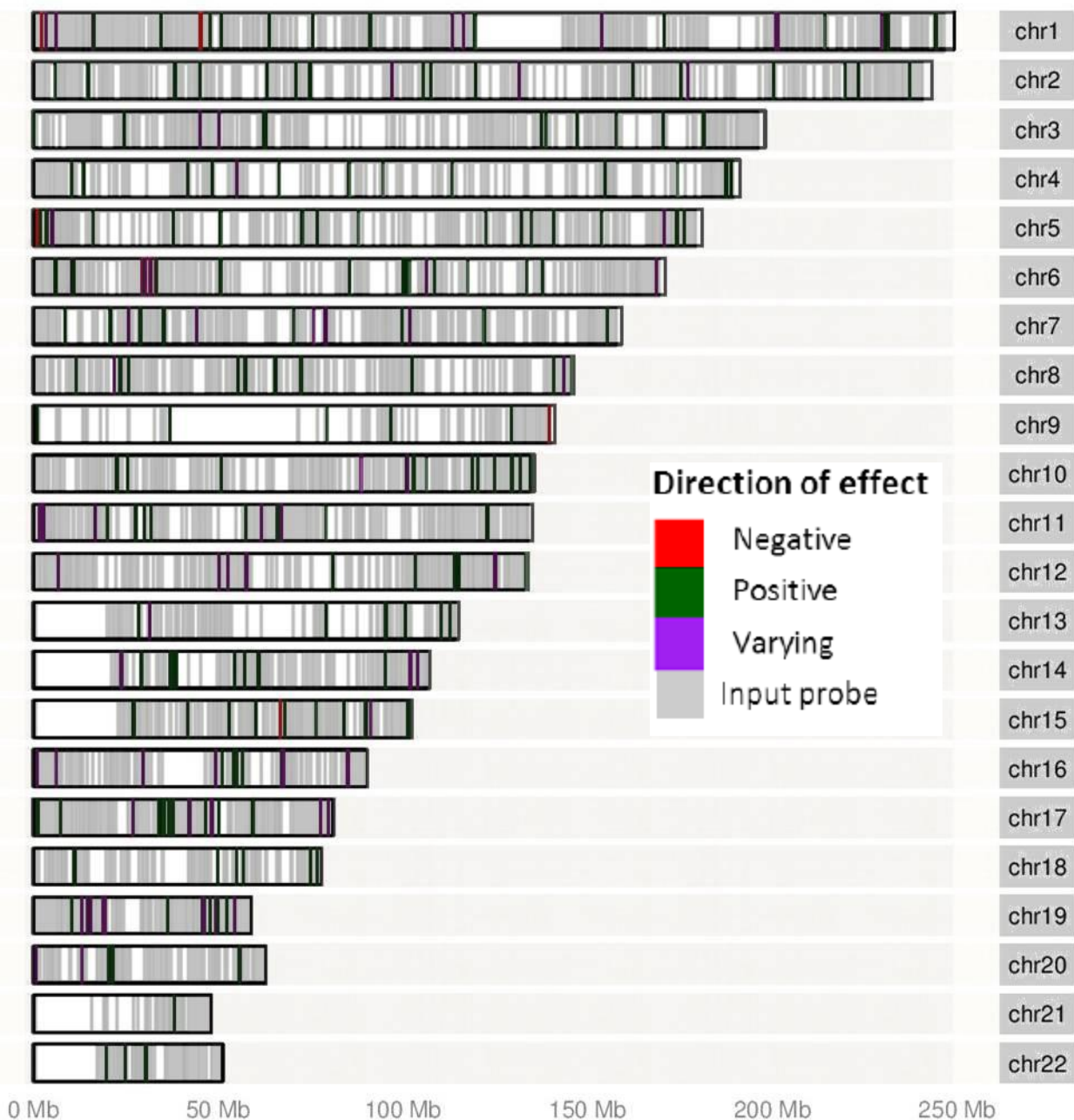

Supplement: Additional file 7: — Autosomal map of age-associated differentially methylated CpG clusters (aDMCs). [file 13148_2015_48_MOESM7_ESM.pdf]
