# Supplementary material for: An epigenetic map of age-associated autosomal loci in northern European families at high risk for the metabolic syndrome
Source: Clin Epigenetics. 2015 Feb 20;7(1):12. doi: 10.1186/s13148-015-0048-6 (PMC4372177; doi:10.1186/s13148-015-0048-6)
Supplement: Additional file 8: — KEGG pathways significantly enriched in positive aDMCs identified by the FatiGO analysis. [file 13148_2015_48_MOESM8_ESM.docx]

**Additional File 8. KEGG pathways enriched in aDMCs identified by the FatiGO analysis**

| #term | name | adj_pvalue |
| --- | --- | --- |
| hsa04340 | The Hedgehog signaling pathway | 3.96E-03 |
| hsa04950 | Maturity onset diabetes of the young | 6.26E-03 |
| hsa04080 | Neuroactive ligand-receptor interaction | 1.58E-02 |
